# Supplementary material for: A Salt-Resistant Sodium Carboxymethyl Cellulose Modified by the Heterogeneous Process of Oleate Amide Quaternary Ammonium Salt
Source: Polymers (Basel). 2022 Nov 18;14(22):5012. doi: 10.3390/polym14225012 (PMC9698984; doi:10.3390/polym14225012)
Supplement: Supplementary file 1 [file polymers-14-05012-s001.zip › polymers-2016028-supplementary.pdf]

Attachment 1

Result diagram of ^{1}HNMR hydrogen spectrum characterization

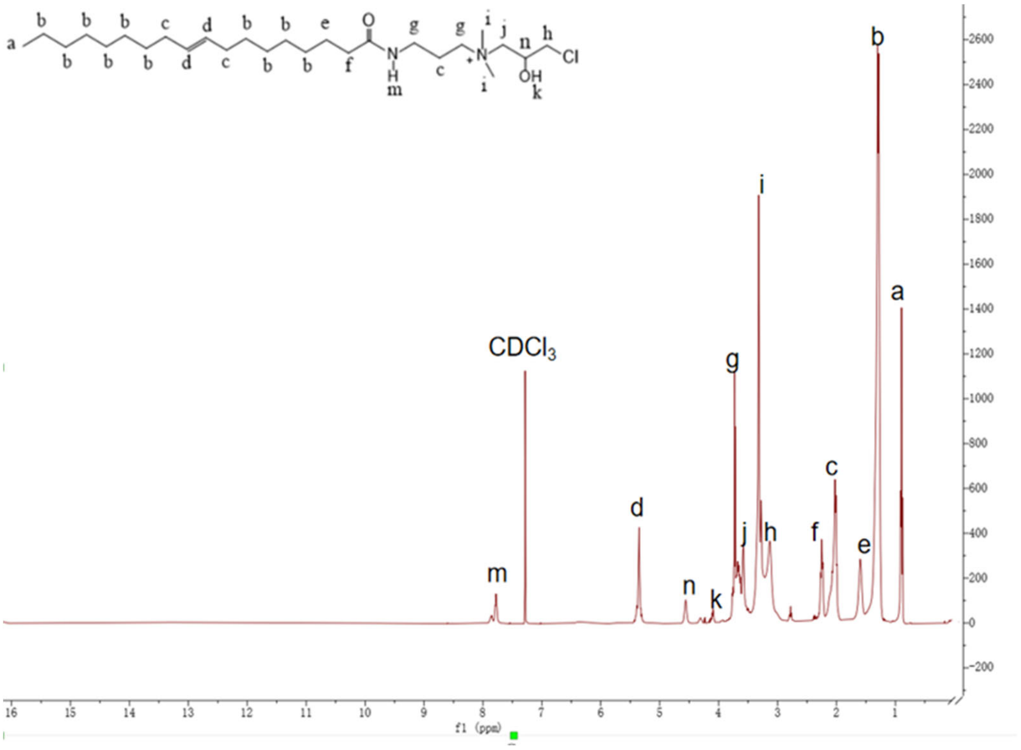

Figure S1. NMR 1H spectrum of oleic acid PKO-ECH intermediate

Shows the ^{1}HNMR (400 MHz, CDCl3) result of intermediate: 0.88[t, 3H, CH_3CH_2], 1.16–1.39 [d, 20H, $CH_3(CH_2)_6CH_2CH$=CH,CH=$CHCH_2(CH_2)_4CH_2$], 1.51–1.67[s,2H,$CH_2CH_2CH_2CO$],1.91-2.18[m,6H,CH_2CH_2CH,$CHCH_2CH_2$,$NHCH_2CH_2CH_2N^+$],2.19–2.33[t,2H,CH_2CH_2CO],3.03–3.20[d,2,CH2CHOH],3.22–3.42[d,6H,$2N^+CH_3$],3.43–3.54[s,2H,N^+CH_2CHOH],3.54–3.85[m,4H,$NHCH_2CH_2CH_2N^+$],3.96–4.11[m,1H,CHOH],4.99–5.11[m,1H,CH_2CHOHCH2], 5.26–5.42 [m, 2H, CH=CH], 7.58–7.76 [br,H, NH][1].

Reference

[1] Mao J, Zhang H, Zhang W, et al. Dissymmetric beauty: A novel design of heterogemini viscoelastic surfactant for the clean fracturing fluid[J]. Journal of industrial and engineering chemistry, 2018, 60: 133-142.
